# An Innovative Strategy for Accurate Thermal Compensation of Gyro Bias in Inertial Units by Exploiting a Novel Augmented Kalman Filter

**DOI:** 10.3390/s18051457

**Published:** 2018-05-07

**Authors:** Rita Fontanella, Domenico Accardo, Rosario Schiano Lo Moriello, Leopoldo Angrisani, Domenico De Simone

**Affiliations:** 1 Department of Industrial Engineering, University of Naples Federico II, Piazzale Tecchio 80, I80125 Naples, Italy; rita.fontanella@unina.it (R.F.); rschiano@unina.it (R.S.L.M.); 2 Department of Electrical Engineering and Information Technology, University of Naples Federico II, Via Claudio 21, I80125 Naples, Italy; angrisan@unina.it; 3 Generale Meccatronica Applicata S.p.A., via Salvatore Piccolo, I80014 Giugliano, Italy; domenico.desimone@gmagroup.it

**Keywords:** Zero Velocity Update, integrated inertial navigation, Kalman filter, MEMS inertial sensors, bias drift, thermal calibration, artificial neural networks

## Abstract

This paper presents an innovative model for integrating thermal compensation of gyro bias error into an augmented state Kalman filter. The developed model is applied in the Zero Velocity Update filter for inertial units manufactured by exploiting Micro Electro-Mechanical System (MEMS) gyros. It is used to remove residual bias at startup. It is a more effective alternative to traditional approach that is realized by cascading bias thermal correction by calibration and traditional Kalman filtering for bias tracking. This function is very useful when adopted gyros are manufactured using MEMS technology. These systems have significant limitations in terms of sensitivity to environmental conditions. They are characterized by a strong correlation of the systematic error with temperature variations. The traditional process is divided into two separated algorithms, i.e., calibration and filtering, and this aspect reduces system accuracy, reliability, and maintainability. This paper proposes an innovative Zero Velocity Update filter that just requires raw uncalibrated gyro data as input. It unifies in a single algorithm the two steps from the traditional approach. Therefore, it saves time and economic resources, simplifying the management of thermal correction process. In the paper, traditional and innovative Zero Velocity Update filters are described in detail, as well as the experimental data set used to test both methods. The performance of the two filters is compared both in nominal conditions and in the typical case of a residual initial alignment bias. In this last condition, the innovative solution shows significant improvements with respect to the traditional approach. This is the typical case of an aircraft or a car in parking conditions under solar input.

## 1. Introduction

In recent years, several innovative solutions for transport systems have been developed, such as Unmanned Aircraft Systems, Unmanned Underwater Systems, Autonomous Ships, Autonomous Land Vehicles, Micro Satellites, and Space Probes [1,2,3,4,5,6,7,8]. These systems have two main features in common, such as the need to exploit compact configurations for the onboard equipment and the capability to perform autonomous operations for a large part of a mission [9]. In addition, new technological frameworks such as the one determined by mechatronics have also risen the interest in developing new motion sensing systems [10]. Indeed, autonomous robot systems used for industrial applications may have significant requirements in terms of localization and attitude determination accuracy [11]. Inertial Measurement Units or IMUs [12] are a reference enabling technology for motion sensing that allows the above reported systems to attain the desired performance in terms of compactness and autonomy.

In recent years, the improvement of advanced micro-fabrication techniques has allowed the development of Micro Electro-Mechanical Systems or MEMS inertial sensors [13]. These systems are manufactured by using the same technology of micro-chips [12]. This peculiarity allows them to be more suitable than traditional inertial sensors to meet compactness and low-power consumption requirements. Also, the specific manufacturing process can assure a valuable cost reduction in large scale applications [14]. The use of IMUs based on MEMS technology improves cost and size performance of more than one order of magnitude with respect to those achievable through Fiber Optic Gyros or FOG. This is the reason why significant effort has been lavished on developing MEMS-based IMUs that can be installed on last-generation transport platforms [15]. The advantage in adopting MEMS-based IMUs can be so relevant in some applications that they are used to retrofit traditional inertial units in already deployed transport systems [16].

MEMS gyros have significant limitations in terms of sensitivity to environmental conditions [17]. They suffer from the change of temperature, since their output average value, i.e., sensor bias, drifts with temperature [17]. The thermal effect, for Coriolis gyros, is caused by decoupling vibrating frequencies on the two axes and by producing distortions on the output of electronic components, such as amplifiers [18]. The typical bias trend model is strongly non-linear with added hysteresis [19]. The traditional approach to compensate this error is to perform calibration tests in a climatic chamber over the requested temperature intervals. The goal of the tests is to derive a calibration function that allows the correction of a large amount of bias. During the real-time operation of the unit, the derived calibration function is applied to correct the sensor output before any other processing step [20]. Subsequently, a further reduction of bias can be realized by estimating its residual amount within a data fusion framework, such as a Kalman filter [21]. This filter has an augmented state, since it includes the bias as a state in addition to errors on attitude, position, and velocity [22,23]. In summary, the traditional approach to estimate gyro bias is a cascading of calibration and Kalman filtering [21]. This approach has several limitations:

(1) It requires extra processing during the real-time operation of units. Since the Kalman filter is needed to keep measurement drift under control, calibration must be added as a pre-processing stage. Moreover, integration of nominal navigation equations have also to be performed, in order to get the uncorrected state derived by standalone inertial measurements. All the above terms have to be provided at an output rate that can reach 1000 measurements per second [24];
(2) It does not account for the deterministic effect of the temperature change on bias drift. This effect is accounted by calibration processing and the residual uncertainty is included in the overall random uncertainty on bias determination. As a consequence, a larger standard deviation than the one needed must be used for modelling process noise on bias inside the Kalman filter [21]. This condition reduces the accuracy on the bias estimated by the filter and increases the settling time.

This paper presents an innovative approach to overcome the limitations mentioned above. The calibration transfer function is exploited within the Kalman filter, with the aim of embedding the compensation of the thermal effect on bias in the filter itself. The time derivative of the thermal effect on bias is estimated as a function both of calibration and time derivative of temperature on the sensor. It is worth noting that this information is made available to users on most inertial units, since it is needed to apply the calibration function. Therefore, the residual uncertainty is substantially reduced so that Kalman filter effectiveness can be improved. Moreover, no calibration processing stage is required, since calibration is performed inside the Kalman filter. This allows the reduction of the computational burden of the IMU processing software. An important advantage over the traditional approach can be appreciated when the gyro bias is affected by hysteresis. As the traditional approach adopts the transfer function as a fixed reference, the presence of hysteresis causes an additive bias. On the contrary, the proposed approach exploits the derivative of the calibration transfer function with temperature. This term tends to be constant on the different lines that form the hysteresis loop [19].

To test the performance of the proposed innovative approach versus the traditional one, a proper testing framework is designed. Testing the system in dynamic conditions can be misleading because the output should be affected by motion-induced errors, such as scale factor error and g-sensitivity error. These errors along with bias and random noise produce a combined effect on navigation state errors and bias tracking capability, which cannot be distinguished from the one of standalone bias because of the intrinsic non-linearity of navigation equations [24]. However, the proposed approach claims a Kalman Filter with sensor data fusion. The best compromise is to use a Kalman Filter that returns meaningful data when the unit is in stationary condition. This is the case of Zero Velocity Update or ZUPT filter. It is a filter used to remove residual errors on navigation state terms and bias at startup for an IMU. It requires that the unit is held fixed with respect to the locally level frame for some minutes [21], since the assumption that the unit is stationary is the aiding information used by the Kalman filter, i.e., the filter measurement model. In stationary condition, any form of linear and angular rate, except the Earth rate, is considered as an error. The ZUPT filter is used to get accurate initial attitude and inertial sensor bias when tactical grade gyros are available, i.e., gyros that have a bias instability that is less than 1 degree per hour [12]. In the case non tactical grade gyros are installed in the IMU, the ZUPT filter is used to estimate just the bias [21]. Indeed, gyro bias cannot be estimated just by averaging sensor output, since the size of averaging time window could not be coherent with thermal variations that induce bias drift. Since thermal variations depend on environmental conditions, nothing is known about their characteristics time. Moreover, when the unit is turned on in stationary condition no information is provided about how long it is going to stay motionless. For this reason, the ZUPT filter has a significant practical interest also for IMUs equipped with standard grade gyros, such as the Attitude and Heading Reference Systems or AHRS installed on aircraft [14] or the Land Navigators installed on ground vehicles [25]. The paper shows how those applications could benefit by exploiting the reported innovative augmented Kalman Filter model.

## 2. Standard ZUPT Filter

### 2.1. Filter Model

**Figure 1:**
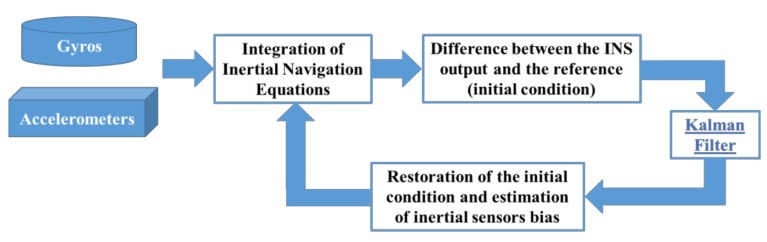
ZUPT filter block diagram model.

The standard ZUPT filter is accurately described in [21]. After a brief coarse alignment phase, the INS attitude and the Position, Velocity, Time (PVT) initial solution are “frozen”. Then the inertial navigation equations are numerically integrated. However, the upgraded attitude and PVT solution are different from their initial values. Since the host vehicle is at rest and non-rotating, changes in attitude and in PVT solution can be due only to uncompensated accelerometers and gyroscopes errors. Measurements of the difference between the INS outputs and the reference are input to a Kalman filter, which corrects the velocity, attitude, and position. Inertial instrument errors, such as accelerometer and gyro biases, are estimated as well. Since ZUPT filter can be applied only when the host vehicle is stationary and non-rotating, it must last only few minutes. Figure 1 shows the block diagram model.

**Figure 2:**
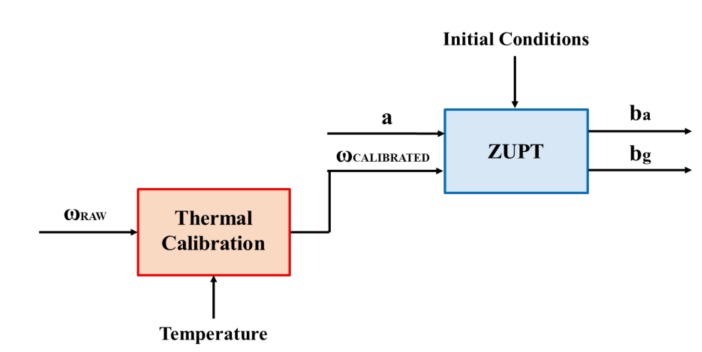
Standard ZUPT filter: a is the accelerometer output, $\omega_{\mathrm{RAW}}$ is the raw gyro data, $\omega_{\mathrm{CALIBRATED}}$ is the calibrated gyro data and $b_{a}$ and $b_{g}$ are respectively the accelerometer and gyro bias.

It is worth noting that the standard ZUPT filter needs calibrated gyro data as input. This is particularly important for MEMS gyros, which are characterized by high levels of noise and poor bias stability characteristics. In particular, MEMS gyro bias has a strong correlation with temperature variations [26,27]. The block diagram model is presented in Figure 2. In the ZUPT algorithm, the filtering step is preceded by thermal calibration of raw gyro data.

In our application, a Back-Propagation Neural Network is used to map the model of the gyro bias trend with temperature. This trend is highly non-linear and it can have a local abrupt change within a small temperature range [26]. Back-Propagation Neural Networks guarantee better performance on mapping than the traditional fitting method based on the application of polynomial fitting [28,29]. In fact, polynomials are not efficient to model these local changes of trend since they have fixed shapes as a function of their order. Instead, Back-Propagation Neural Networks are self-adaptive in constructing a mathematical model after several repetitive learning and testing phases.

### 2.2. Determination of Filter Process Noise Terms

To realize an accurate ZUPT filter, it is necessary to correctly define the elements of $Q_{k}$, which is the 12 × 12 covariance matrix of the process noise, whose elements are defined as follows [21]:

- $Q_{k}(1,1),Q_{k}(2,2),Q_{k}(3,3)$ are related to the accelerometer measurement noise ${\vec{\eta }}_{a}$.
  (1) $$Q_{k}(1,1)=Q_{k}(2,2)=Q_{k}(3,3)=\sigma_{VRW}^{2}$$
  where $\sigma_{VRW}^{}$ is the velocity random walk of the accelerometer measurement.
- $Q_{k}(4,4),Q_{k}(5,5),Q_{k}(6,6)$ are related to the gyro measurement noise ${\vec{\eta }}_{g}$.
  (2) $$Q_{k}(4,4)=Q_{k}(5,5)=Q_{k}(6,6)=\sigma_{ARW}^{2}$$
  where $\sigma_{ARW}^{}$ is the angular random walk of the gyro measurement.>
- $Q_{k}(7,7),Q_{k}(8,8),Q_{k}(9,9)$ are related to the accelerometer bias ${\vec{\eta }}_{ba}$.
  (3) $$Q_{k}(7,7)=Q_{k}(8,8)=Q_{k}(9,9)=\sigma_{ABI}^{2}$$
  where $\sigma_{ABI}^{}$ is the bias instability of the accelerometer measurement.
- $Q_{k}(10,10),Q_{k}(11,11),Q_{k}(12,12)$ are related to the gyro bias ${\vec{\eta }}_{bg}$.
  (4) $$Q_{k}(10,10)=Q_{k}(11,11)=Q_{k}(12,12)=\sigma_{GBI}^{2}$$
  where $\sigma_{GBI}^{}$ is the bias instability of the gyro measurement.

The off-diagonal elements of $Q_{k}$ are zero ($Q_{k}$ is a diagonal matrix). The diagonal elements of $Q_{k}$ have been evaluated through the Allan Variance analysis.

The Allan Variance is a simple and efficient method to identify and characterize different stochastic processes and their coefficients, allowing estimation of the accidental component of errors that affect the signal [30,31]. Through some simple operations on the sensor outputs, a characteristic Allan Variance curve can be obtained and further used to determine the type and magnitude of errors affecting the sensor data [32]. If *N* is the number of samples from an inertial sensor with a sample time τ*_0_*, a group of *n* data points can be created (with *n* < *N*/2); each group member is called a cluster τ with size *n*τ*_0_*. If Ω (*t*) is the instantaneous output of the sensor, its integration (e.g., for the gyro output) is the angle θ (*t*) [33]:

(5) $$\theta (t)=\int_{t}\Omega (t)dt$$

The angle is measured at discrete times given by *t = kτ_0_* (for *k* = 1, 2, 3, ..., *N*). By using the notation *θ*(*t*) = *θ*(*kτ_0_*) = *θ_k_*, the average angle between the times *kτ_0_* and (*kτ_0_* + *τ*) is given by [33]:

(6) $${\bar{\theta }}_{k}\left(\tau \right)=\frac{1}{\tau }\overset{k\tau_{0}+\tau }{\underset{k\tau_{0}}{\int }}\Omega \left(t\right)dt,\;\;\;\tau =n\tau_{0}$$

The Allan Variance, estimated from a finite number of samples, is defined as follows [33]:

(7) $$\sigma^{2}\left(\tau \right)=\frac{1}{2\tau^{2}\left(N-2n\right)}\overset{N-2n}{\underset{n=1}{\sum }}{\left(\theta_{k+2n}-2\theta_{k+n}+\theta_{k}\right)}^{2}$$

The most attractive feature of this method is the ability to define various error components by the slope of the root Allan Variance (i.e., the Allan deviation) plot. Typical errors affecting inertial sensors, which are detectable through the Allan Variance, are the quantization noise, angle random walk, correlated noise, sinusoidal noise, bias instability, rate random walk, and rate ramp. Correlated and sinusoidal noises have minor contributions to the total noise, and they appear only at long-time clusters; all the other errors are believed to have the most impact on MEMS sensors [34].

**Figure 3:**
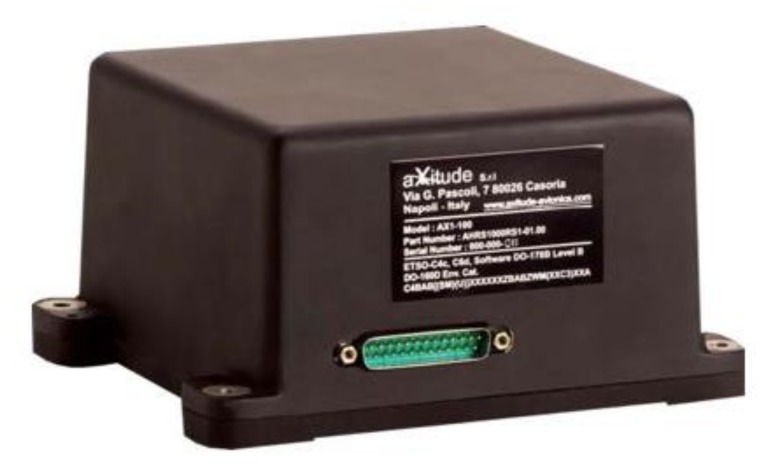
Axitude AX1-[ ]^TM^.

In the proposed application, the Attitude and Heading Reference System Axitude AX-[ ]^TM^ has been used, that is depicted in Figure 3. This device is composed by the following sensors [35]:

- Triaxial accelerometer sensor;
- Triaxial gyroscope sensor;
- Triaxial magnetometer sensor;
- Temperature sensor.

The adopted gyroscopes are the CRS05-02^TM^ gyros by Silicon Sensing^TM^ (Plymouth, UK) while the adopted accelerometers are the MS8010^TM^ accelerometers by Colybris^TM^ (Yverdon-les-Bains, Switzerland). The error components of interest in our application, obtained by the slope of the Allan deviation plot, are shown in Table 1 and Table 2.

## 3. Modified ZUPT Filter

**Figure 4:**
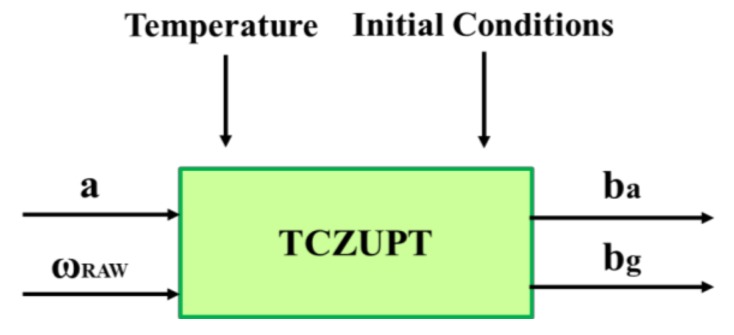
TCZUPT filter: a is the accelerometer output, $\omega_{\mathrm{RAW}}$ is the raw gyro data and $b_{a}$ and $b_{g}$ are respectively the accelerometer and gyro bias.

As explained in Section 2.1, the traditional process is time consuming and expensive, since the ZUPT filter needs thermal calibrated gyro data as input. This paper proposes an innovative ZUPT filter, i.e., the Thermal Compensated ZUPT (TCZUPT) filter, that needs raw gyro data as input by unifying the two steps of thermal calibration and filtering (Figure 4).

The proposed method assumes a different model for the gyro bias with respect to the standard ZUPT filter, in which the bias time derivative is modelled as a zero-mean Gaussian noise:

(8) $${\dot{\vec{b}}}_{gZUPT}(t)={\vec{\eta }}_{bg}(t)$$

where ${\vec{\eta }}_{bg}(t)$ is a zero-mean Gaussian noise.

In the TCZUPT filter, instead, it is modelled as the combination of two terms, such as a temperature dependent component and a stochastic component:

(9) $${\dot{\vec{b}}}_{gTCZUPT}(t)=\frac{d{\vec{b}}_{g}(T(t))}{dt}+\vec{\eta }{'}_{bg}(t)$$

where $\vec{\eta }{'}_{bg}(t)$ is the stochastic component, modelled as a zero-mean Gaussian noise ($\vec{\eta }{'}_{bg}(t)$ < ${\vec{\eta }}_{bg}(t)$ of Equation (8)) and $\frac{d{\vec{b}}_{g}(T(t))}{dt}$ is the temperature dependent component, defined as follows:

(10) $$\frac{d{\vec{b}}_{g}(T(t))}{dt}=\frac{\partial {\vec{b}}_{g}}{\partial T}\cdot \frac{dT}{dt}$$

where $\frac{\partial {\vec{b}}_{g}}{\partial T}$ is the partial derivative of gyro bias with respect to temperature and $\frac{dT}{dt}$ is the time derivative of temperature. These two terms can be obtained as follows:

- Back-Propagation Neural Networks are used to estimate the derivative of gyro bias with respect to temperature.
- The algorithm proposed in [36] is used to evaluate the time derivative of temperature data obtained by the temperature sensor inside the AHRS. In every time-point, it takes into account the time-history of the derivative.

Therefore, in the TCZUPT filter, the gyro bias propagation in the prediction step is:

(11) $$b_{g,\;\;k}^{-}=\left(I_{3x3}+F_{g,k}\cdot T_{s}\right)\cdot b_{g,k-1}+\frac{db_{g,k}}{dt_{k}}\cdot T_{s}$$

where $I_{3x3}$ is the 3 × 3 identity matrix, $F_{g,k}$ is the 3 × 3 null matrix, *T_s_* is the sample period, $\;\frac{db_{g,k}}{dt_{k}}$ is the discrete-time derivative of gyro bias and $\eta {'}_{bg,k}\;$ is the zero-mean Gaussian noise.

Also in the TCZUPT filter, it is necessary to correctly define the elements of the covariance matrix of the process noise $Q_{k}$ (Equations (1)–(4)). The accelerometer and gyro error components are presented in Table 3 and Table 4. It is worth noting that the velocity random walk $\sigma_{VRW}^{}$ and accelerometer bias instability $\sigma_{ABI}^{}$ are the same of the standard ZUPT filter, as well as the gyro angular random walk $\sigma_{ARW}^{}$. The only different term is the gyro bias instability $\sigma_{GBI}^{}$, since the TCZUPT filter receives as input raw gyro data, which have a higher bias instability than the calibrated data used in the standard ZUPT filter.

In conclusion, it is worth noting that the TCZUPT filter requires a simplified configuration with respect to the standard ZUPT filter, which needs two processing phases, one for thermal calibration and the other for filtering.

In this paper, the proposed model has been implemented in the error-states Kalman Filter. However, it is worth noting that it can be also valid in the total-states approach. Ref. [37] describes the different fusion schemes and their performance. In many applications, it is convenient to use a total-states instead of the error states Kalman Filter.

## 4. Experimental Test Setup

Two thermal tests have been performed to determine MEMS gyro bias under different temperature points. These tests have been carried out at the laboratory of the Generale Meccatronica Applicata (G.M.A.), located in Giugliano in Campania (Italy). As described in Section 2, the AHRS used in our application is the Axitude AX1-[ ]^TM^ (Figure 3).

The test equipment used to perform thermal tests consists of the following components:

- A Heraus-HT7057 climatic chamber, which has a nominal temperature range from −70 °C to +180 °C;
- A power supply for the AHRS (24–28 V);
- A personal computer with a RS-232 interface connected to the AHRS and the climatic chamber;
- A software data logger that communicates with the AHRS through the serial interface 232, using a Baud Rate of 115,200 bps;
- Two external temperature sensors, one attached to the device, the other attached to the inner wall of the climatic chamber. These supplementary sensors are used to control temperature variations during the tests;
- A data acquisition/switch unit connected to another PC to acquire measurements from the two supplementary sensors.

**Figure 5:**
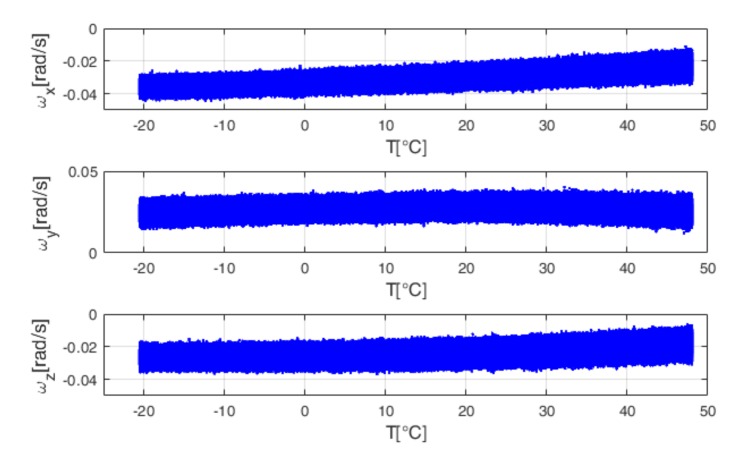
Gyro output ($\omega_{x},\;\omega_{y},\omega_{z}$) vs. temperature for the soak test.

**Figure 6:**
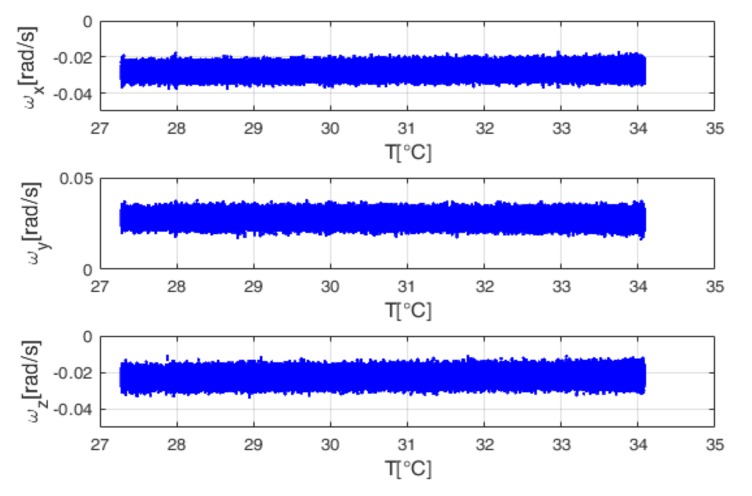
Gyro output ($\omega_{x},\;\omega_{y},\omega_{z}$) vs. temperature for the ramp test.

The inertial unit is installed inside the climatic chamber in stationary conditions. This chamber allows for performing controlled thermal solicitations on the unit by assigning proper temperature profiles. Two thermal tests have been performed:

- The first is a soak test, where gyro temperature varies from −20.53 °C to 48.08 °C, with steps of 5 °C. In this test, the gyro temperature is continuously stabilized at certain temperature points [17]. The soak time for each temperature point is two hours and a half. Figure 5 shows the output trend with temperature.
- The second is a ramp test, where gyro temperature varies from 27.28 °C to 34.08 °C. In this test, the temperature of the thermal chamber is continuously linearly increased or decreased, without stabilizing the gyro temperature at certain temperature points [17]. The ramp rate is 2 °C per minute. This is a typical condition for gyros installed on the AHRS considered in this paper, which is generally used for aeronautical applications. Figure 6 shows the output trend with temperature, for the ramp test.

The AHRS sensor data are transmitted as packages of data on a fixed binary format over a RS232 serial communication interface [35]. In this process, temperature is the reference environmental term considered. Temperature and static gyro output are measured in real-time to observe the bias drift phenomenon when temperature variations are commanded. It is worth noting that both data sets have been filtered by using the low-pass filter usually adopted in avionic certified AHRS versions to remove out of band noise. It is a second order linear filter with cutoff frequency of 30 Hz.

## 5. Derivation of Calibration Function

Data obtained from the soak test are used to train the Back-Propagation Neural Network on a wide temperature range, whereas data obtained from the ramp test are used to test the ZUPT and TCZUPT filter. These filters have both been coded by using MATLAB^TM^ (Natick, MA, USA). The MATLAB Neural Network toolbox^TM^ helps to assess the best Back-Propagation Neural Network structure that can model the bias or bias derivative evolution versus temperature for each specific gyro. In this paper, Back-Propagation Neural Networks have been trained by using the Levenberg-Marquardt algorithm [38]. It is an iterative three-step process including training, validating, and testing. This algorithm needs to share the dataset among three different uniformly distributed subsets, such as:

- 65% of samples for training;
- 20% of samples for validating;
- 15% of samples for testing.

To select the most suitable Neural Network structure, we have taken into account the theorem presented in [39]. According to this theorem an adequate solution can be obtained with a tractable network size by using more than three layers [39]. In our application, Back-Propagation Neural Networks composed by four layers (an input layer, two hidden layers and an output layer) have been implemented.

To select the number of hidden layer neurons, the performance parameter *Sr*, defined in Equation (12), has been evaluated [29]. The same number of processing neurons has been selected for the two hidden layers, since this is the minimum condition for proper use of network:

(12) $$Sr\;\left(n\right)=\sqrt{res{\left(n\right)}_{1}^{2}+\;res{\left(n\right)}_{2}^{2}+res{\left(n\right)}_{3}^{2}}$$

In Equation (12), $res{\left(n\right)}_{1},\;res{\left(n\right)}_{2}\;$ and $res{\left(n\right)}_{3}$ are the residuals for the triaxial gyroscope sensor, for a generic Neural Network composed by two *n-*neurons hidden layers.

The standard ZUPT filter needs calibrated gyro data as input. Therefore, the developed Back-Propagation Neural Network has been used to calibrate the testing data sets presented in Figure 6 (obtained by the ramp test), before the filtering process. In the TCZUPT filter, instead, thermal calibration and filtering are simultaneously performed. Therefore, it needs raw gyro data as input. During the execution of the filtering process, Back-Propagation Neural Networks trained on the derivative of the training data sets presented in Figure 5 are used to estimate the derivative with respect to temperature of the testing data sets presented in Figure 6.

**Figure 7:**
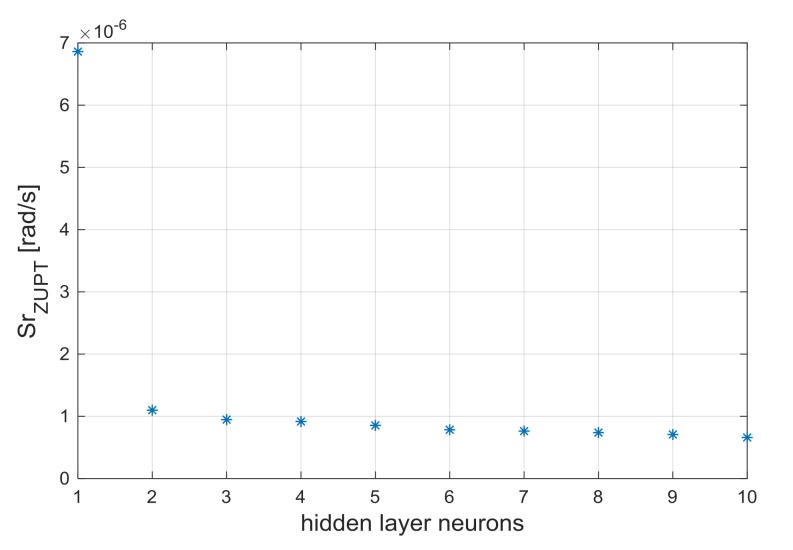
Performance parameter Sr_ZUPT_ vs. number of hidden layers neurons (standard ZUPT filter).

**Figure 8:**
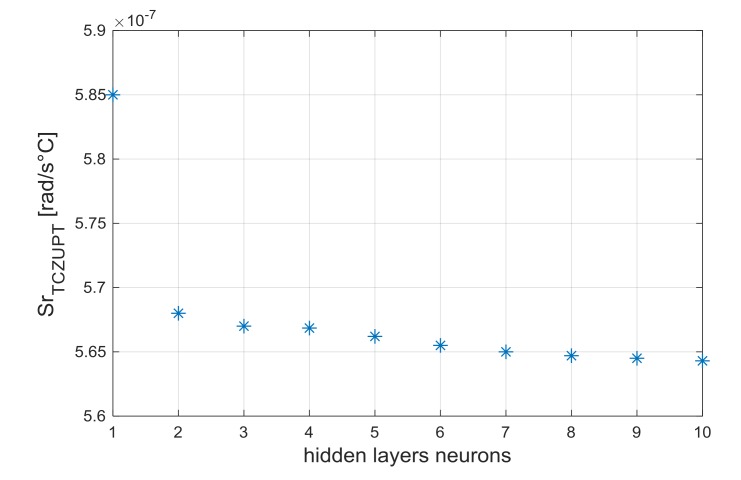
Performance parameter Sr_TCZUPT_ vs. number of hidden layers neurons (TCZUPT filter).

In the standard ZUPT and TCZUPT filter, Back-Propagation Neural Networks with two hidden layers of two neurons have been used. From Figure 7 and Figure 8, which present the trend of the performance parameter with the number of hidden layers neurons, it is evident that the selected structures can provide a satisfactory convergence effect in both methods.

## 6. ZUPT Filter Comparative Performance Analysis

**Figure 9:**
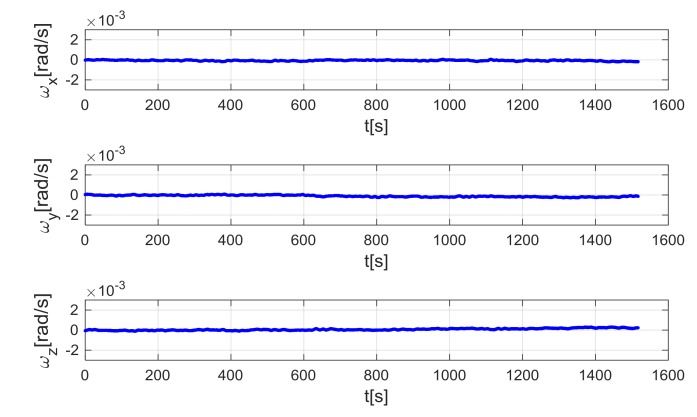
Input data of the standard ZUPT filter.

**Figure 10:**
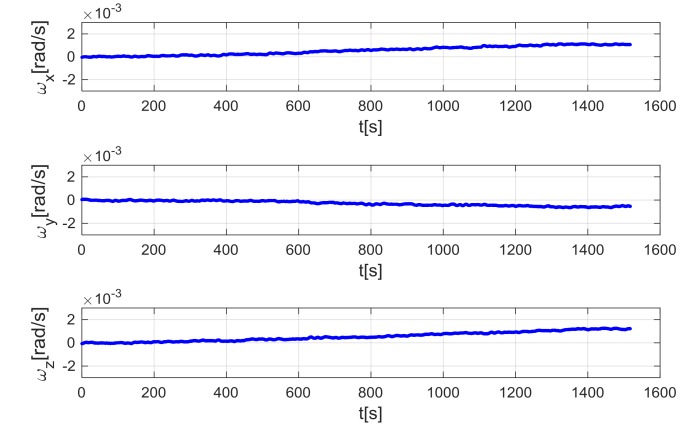
Input data of the TCZUPT filter.

Gyro data obtained by the ramp test have been used to test both the standard ZUPT and TCZUPT filter. However, as explained in the previous sections, the standard ZUPT filter requires calibrated gyro data as input, whereas the TCZUPT filter needs raw gyro data. Figure 9 and Figure 10 show the input data of the standard ZUPT and TCZUPT filter. To highlight the different trends of the two data sets, a second order low-pass filter with cutoff frequency of 1 Hz has been used to remove out of band noise in representing data. However, it is worth noting that the ZUPT filter has been tested on data filtered by using the second order low-pass filter with cutoff frequency of 30 Hz, which is usually adopted in avionic certified AHRS versions. In both cases, before applying the ZUPT filter, the initial zeroing procedure has been performed in a time-frame of 60 s.

To compare the standard ZUPT and TCZUPT filter performance, a benchmark must be defined. In this application, the considered benchmark is the moving average of raw and calibrated gyro data. This processing step returns an array of local mean values, where each mean is calculated over a sliding window of 30 s across neighboring elements of the input vector. Table 5 presents the mean of residuals of the moving averages, which can be considered an estimator of the true bias of the system, with an accuracy given by the corresponding residual. The level of residuals is adequate to justify their use as a reference benchmark for the filter.

**Figure 11:**
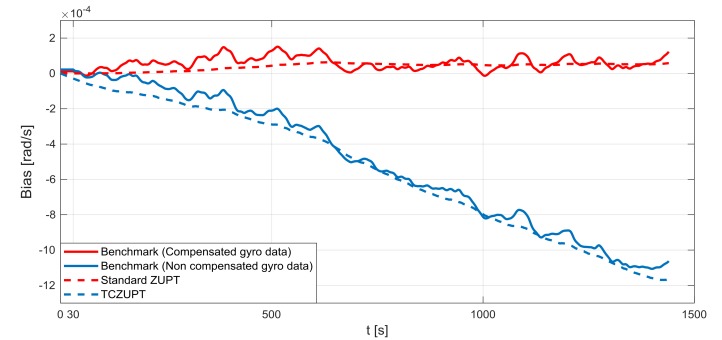
Gyro output bias (nominal condition, *x*-axis).

**Figure 12:**
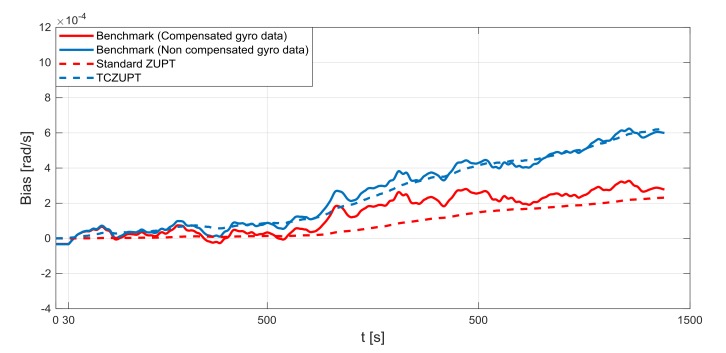
Gyro output bias (nominal condition, *y*-axis).

**Figure 13:**
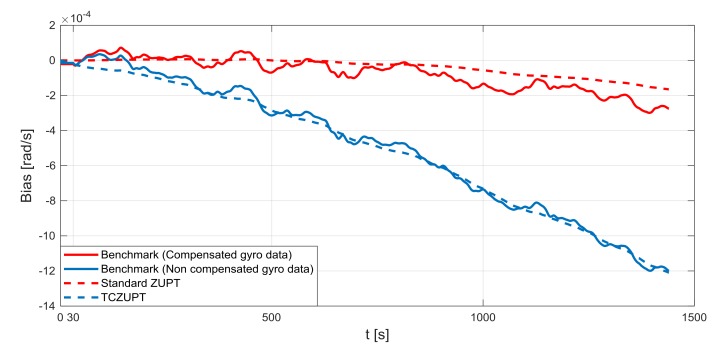
Gyro output bias (nominal condition, *z*-axis).

**Figure 14:**
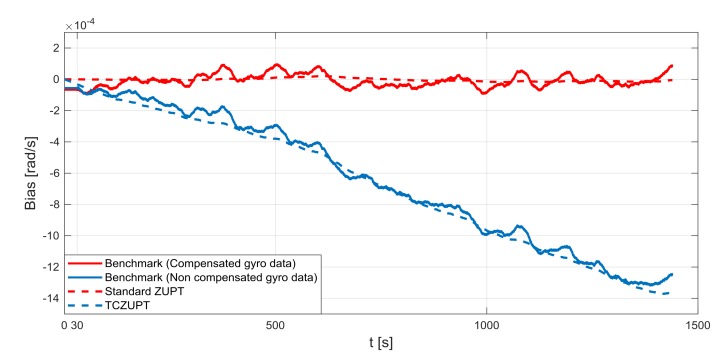
Gyro output bias (residual error of 15 degree/h, *x*-axis).

**Figure 15:**
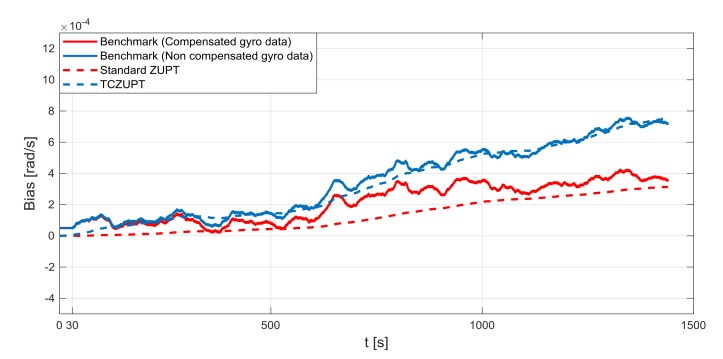
Gyro output bias (residual error of 15 degree/h, *y*-axis).

**Figure 16:**
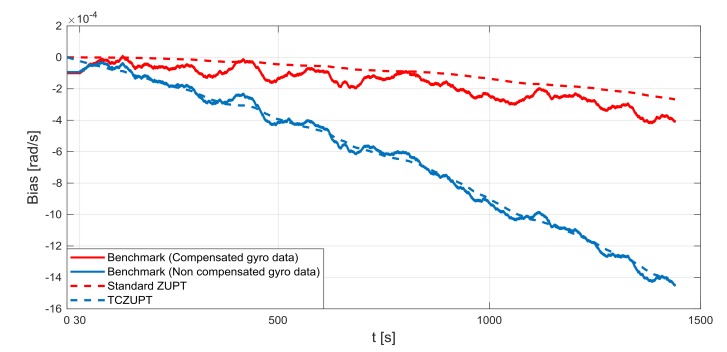
Gyro output bias (residual error of 15 degree/h, *z*-axis).

The standard ZUPT and TCZUPT filter have been tested under two sets of conditions:

- Nominal condition. Figure 11, Figure 12 and Figure 13 present the comparison of the true bias of the system, estimated by the moving averages and the bias computed by the standard ZUPT and TCZUPT filter. Table 6 presents the root mean squared error (rms) of both methods, in the initial 30 s and over the entire interval.
- Residual bias after rough initial alignment of 15 degree/h. Since the considered gyro is not tactical grade, this residual error is compatible with its performance. Figure 14, Figure 15 and Figure 16 present the comparison of the true bias of the system, estimated by the moving averages and the bias computed by the standard ZUPT and TCZUPT filter. Table 7 presents the root mean squared error (rms) of both methods, in the initial 30 s and over the entire interval.

To assess the statistical significance of the variations between the results for the x, y, and z axes, the testing data set has been divided in 5 data sets corresponding to 5 min of acquisition. Then, the ZUPT and TCZUPT filters have been tested on these data sets. Table 8 presents the root mean squared error (rms) of both methods in nominal conditions and Table 9 shows the mean and standard deviation of the results. Table 10 presents the root mean squared error (rms) of both methods in case of a residual initial alignment error of 15 degree/h and Table 11 shows the mean and standard deviation of the results. ZUPT and TCZUPT filters performance is slightly different for the three axes, since each gyro has different characteristics, even if all of the three gyros are of the same type (CRS05-02^TM^ gyros by Silicon Sensing^TM^). However, the small values of the standard deviation (Table 9 and Table 11) indicate that, for each axis, the performance of the ZUPT and TCZUPT filters is stable.

As reported by Table 7, in the first 30 s the rms error of the TCZUPT filter is smaller than the corresponding value of the standard ZUPT filter. Indeed, in the case of a residual initial alignment error of 15 degree/h both methods converge to the true bias of the system, but the TCZUPT filter is faster. This is due to the TCZUPT filter is not based on direct correction of thermal bias (as the standard ZUPT filter), but on the estimation of the derivative of bias as a function of temperature. Therefore, it is faster in mapping significant bias variations. Table 12 presents the convergence time of the standard ZUPT and TCZUPT filter in case of residual error of 15 degree/h. A threshold of 1.00 × 10^−05^ rad/s has been considered in defining the convergence time.

This is an important result for many applications that require a very fast ZUPT process, like missile systems. The TCZUPT filter also presents better performance in the overall interval. This result can be useful in the case of stationary condition for long timeframe, e.g., the aircraft in parking stall for a time longer than the usual in a sunny day.

It is worth noting that, although the standard ZUPT and the TCZUPT filters have been tested on a MEMS-based IMU, also IMUs based on FOG technology can benefit from the advantages of TCZUPT filter. These higher-grade gyros provide high-precision information for navigation and control systems. Thus, they can be used for a wide range of tactical and commercial applications, such as Unmanned Underwater Vehicles (UUVs) and Unmanned Air Vehicles (UAVs), torpedoes, camera and antenna stabilization, land navigation, AHRS, gyrocompasses, and oil drilling [40]. However, IMUs based on FOG technology are subjected to various environmental disturbances. In particular, temperature variation is the major factor that affects FOG performance [41]. Several research efforts have been made to reduce this effect [41,42,43,44,45]. Since TCZUPT filter can be directly applied on raw data, it allows to simplify the management of thermal correction process also for IMUs based on FOG technology, saving time and economic resources.

An important advantage of the proposed procedure is that only a single software component is needed to estimate the attitude during real-time operation of the unit, i.e., the TCZUPT algorithm. In standard operation, the processing schedule requires that two different components must operate in cascading conditions, such as pointwise calibration software and standard ZUPT filter. Of course, this type of architecture has a negative impact on software development and maintenance. Moreover, the TCZUPT algorithm operates on raw sensor data so that no further modification can be performed that changes error terms before applying the filter. In the standard ZUPT filter, the change of calibration method would determine modifications that could alter the filter performance, such as modification of the spectral distribution of correction.

In the case of nominal conditions, the standard ZUPT and TCZUPT filter have similar performance. Indeed, it is worth noting from Figure 11, Figure 12 and Figure 13 that both methods converge to the true bias of the system. The bias estimated by the standard ZUPT and TCZUPT filter respectively converge to the bias of calibrated gyro output and non-calibrated gyro output.

## 7. Conclusions

This paper presents an innovative model for integrating thermal compensation of gyro bias error into an augmented state Kalman filter to improve performance of inertial units manufactured by exploiting MEMS gyros. Despite the advantages of low-cost, light-weight, high reliability and low power consumption, MEMS gyros are characterized by a strong correlation of the systematic error with temperature variations. Thus, the traditional ZUPT filter should be preceded by thermal calibration of raw gyro data. However, this process is time consuming and expensive.

This paper proposes an innovative method that unifies the two steps of thermal calibration and filtering, so that the ZUPT filter can be directly applied on raw gyro data. The main difference with respect to the standard method is the gyro bias time-model. In the standard ZUPT filter, it is modelled as a zero-mean Gaussian noise, instead, in the TCZUPT filter, it is modelled as the composition of two terms: a temperature dependent component and a stochastic component, which is modelled as a zero-mean Gaussian noise.

The analytical description as well as the comparison of the standard ZUPT and TCZUPT filters are presented in the paper. Also, the process to determine the filter terms and the experimental test setup are accurately described. The performance of the two filters is compared in nominal conditions and in the case of a residual error of 15 degree/h. In the case of a residual error of 15 degree/h, the TCZUPT filter guarantees a faster convergence to the true bias of the system and better performance in the overall interval. Furthermore, it is possible to notice that the TCZUPT filter requires a simplified configuration with respect to the standard ZUPT filter, which needs two processing stages, one for thermal calibration and the other for filtering. Thus, the TCZUPT filter allows simplifying the real-time processing of inertial systems by exploiting a single processing function.

## Figures and Tables

**Table 1:** Angular random walk $\sigma_{ARW}^{}$ and gyro bias instability $\sigma_{GBI}^{}$ (TCZUPT filter).

CRS05-02^TM^ Gyro by Silicon Sensing^TM^	
$\sigma_{ARW}^{}$	$\sigma_{GBI}^{}$
3.18 degree/√h	1.5 × 10^−4^ degree/s

**Table 2:** Velocity random walk $\sigma_{VRW}^{}$ and accelerometer bias instability $\sigma_{ABI}^{}$ (standard ZUPT filter).

MS8010^TM^ Accelerometer by Colybris^TM^	
$\sigma_{VRW}^{}$	$\sigma_{ABI}^{}$
0.05 m/(s√h)	2 m/(sh)

**Table 3:** Angular random walk $\sigma_{ARW}^{}$ and gyro bias instability $\sigma_{GBI}^{}$ (TCZUPT filter).

CRS05-02^TM^ Gyro by Silicon Sensing^TM^	
$\sigma_{ARW}^{}$	$\sigma_{GBI}^{}$
3.18 degree/√h	5 × 10^−4^ degree/s

**Table 4:** Velocity random walk $\sigma_{VRW}^{}$ and accelerometer bias instability $\sigma_{ABI}^{}$ (TCZUPT filter).

MS8010^TM^ Accelerometer by Colybris^TM^	
$\sigma_{VRW}^{}$	$\sigma_{ABI}^{}$
0.05 m/(s√h)	2 m/(sh)

**Table 5:** Mean value of residuals of the moving average.

Axis	Calibrated Gyro Data	Non-Calibrated Gyro Data
*x*	1.68 × 10^−6^ rad/s	9.53 × 10^−6^ rad/s
*y*	2.98 × 10^−6^ rad/s	6.80 × 10^−6^ rad/s
*z*	3.43 × 10^−6^ rad/s	1.03 × 10^−5^ rad/s

**Table 6:** Root Mean Squared Error (nominal conditions).

Initial 30 s		
Axis	Standard ZUPT Filter	TCZUPT Filter
x	1.24 × 10^−5^ rad/s	3.78 × 10^−5^ rad/s
y	3.17 × 10^−5^ rad/s	3.46 × 10^−5^ rad/s
z	2.11 × 10^−5^ rad/s	7.88 × 10^−6^ rad/s
**Entire Interval**		
**Axis**	**Standard ZUPT Filter**	**TCZUPT Filter**
*x*	4.15 × 10^−5^ rad/s	5.33 × 10^−5^ rad/s
*y*	7.48 × 10^−5^ rad/s	2.97 × 10^−5^ rad/s
*z*	6.07 × 10^−5^ rad/s	3.30 × 10^−5^ rad/s

**Table 7:** Root Mean Squared Error (residual error of 15 degree/h).

Initial 30 s		
Axis	Standard ZUPT Filter	TCZUPT Filter
*x*	6.66 × 10^−5^ rad/s	4.09 × 10^−5^ rad/s
*y*	5.07 × 10^−5^ rad/s	4.60 × 10^−5^ rad/s
*z*	1.01 × 10^−4^ rad/s	8.16 × 10^−5^ rad/s
**Entire Interval**		
**Axis**	**Standard ZUPT Filter**	**TCZUPT Filter**
x	3.98 × 10^−5^ rad/s	4.13 × 10^−5^ rad/s
y	9.72 × 10^−5^ rad/s	3.88 × 10^−5^ rad/s
z	8.17 × 10^−5^ rad/s	3.01 × 10^−5^ rad/s

**Table 8:** Root Mean Squared Error (nominal conditions, 5 data sets).

Data Set	Axis	Standard ZUPT Filter	TCZUPT Filter
1st	*x*	4.25 × 10^−5^ rad/s	5.35 × 10^−5^ rad/s
	*y*	2.65 × 10^−5^ rad/s	1.96 × 10^−5^ rad/s
	*z*	5.03 × 10^−5^ rad/s	4.48 × 10^−5^ rad/s
2nd	*x*	2.63 × 10^−5^ rad/s	4.53 × 10^−5^ rad/s
	*y*	3.17 × 10^−5^ rad/s	2.12 × 10^−5^ rad/s
	*z*	4.89 × 10^−5^ rad/s	4.74 × 10^−5^ rad/s
3rd	*x*	2.99 × 10^−5^ rad/s	3.91 × 10^−5^ rad/s
	*y*	5.03 × 10^−5^ rad/s	3.43 × 10^−5^ rad/s
	*z*	3.76 × 10^−5^ rad/s	3.81 × 10^−5^ rad/s
4th	*x*	4.26 × 10^−5^ rad/s	4.44 × 10^−5^ rad/s
	*y*	3.54 × 10^−5^ rad/s	3.17 × 10^−5^ rad/s
	*z*	4.94 × 10^−5^ rad/s	3.28 × 10^−5^ rad/s
5th	*x*	2.99 × 10^−5^ rad/s	5.29 × 10^−5^ rad/s
	*y*	3.53 × 10^−5^ rad/s	2.48 × 10^−5^ rad/s
	*z*	3.21 × 10^−5^ rad/s	3.16 × 10^−5^ rad/s

**Table 9:** Mean and Standard Deviation (nominal conditions, 5 data sets).

	Axis	Mean	Standard Deviation
**Standard ZUPT Filter**
	*x*	3.42 × 10^−5^ rad/s	0.77 × 10^−5^ rad/s
	*y*	3.58 × 10^−5^ rad/s	0.89 × 10^−5^ rad/s
	*z*	4.36 × 10^−5^ rad/s	0.82 × 10^−5^ rad/s
**TCZUPT Filter**	**Axis**	**Mean**	**Standard Deviation**
	*x*	4.70 × 10^−5^ rad/s	0.61 × 10^−5^ rad/s
	*y*	2.63 × 10^−5^ rad/s	0.65 × 10^−5^ rad/s
	*z*	3.89 × 10^−5^ rad/s	0.70 × 10^−5^ rad/s

**Table 10:** Root Mean Squared Error (residual error of 15 degree/h, 5 data sets).

Data Set	Axis	Standard ZUPT Filter	TCZUPT Filter
1st	*x*	6.27 × 10^−5^ rad/s	4.11 × 10^−5^ rad/s
	*y*	8.08 × 10^−5^ rad/s	4.61 × 10^−5^ rad/s
	*z*	7.49 × 10^−5^ rad/s	3.27 × 10^−5^ rad/s
2nd	*x*	5.95 × 10^−5^ rad/s	3.27 × 10^−5^ rad/s
	*y*	8.88 × 10^−5^ rad/s	5.14 × 10^−5^ rad/s
	*z*	7.42 × 10^−5^ rad/s	3.84 × 10^−5^ rad/s
3rd	*x*	5.37 × 10^−5^ rad/s	3.18 × 10^−5^ rad/s
	*y*	1.02 × 10^−4^ rad/s	5.43 × 10^−5^ rad/s
	*z*	6.35 × 10^−5^ rad/s	3.12 × 10^−5^ rad/s
4th	*x*	7.27 × 10^−5^ rad/s	4.14 × 10^−5^ rad/s
	*y*	7.94 × 10^−5^ rad/s	3.25 × 10^−5^ rad/s
	*z*	6.36 × 10^−5^ rad/s	3.00 × 10^−5^ rad/s
5th	*x*	7.60 × 10^−5^ rad/s	4.33 × 10^−5^ rad/s
	*y*	9.31 × 10^−5^ rad/s	4.49 × 10^−5^ rad/s
	*z*	8.18 × 10^−5^ rad/s	3.60 × 10^−5^ rad/s

**Table 11:** Mean and Standard Deviation (residual error of 15 degree/h, 5 data sets).

	Axis	Mean	Standard Deviation
**Standard ZUPT Filter**
	*x*	6.49 × 10^−5^ rad/s	0.92 × 10^−5^ rad/s
	*y*	8.88 × 10^−5^ rad/s	0.92 × 10^−5^ rad/s
	*z*	7.16 × 10^−5^ rad/s	0.79 × 10^−5^ rad/s
**TCZUPT Filter**	**Axis**	**Mean**	**Standard Deviation**
	*x*	3.81 × 10^−5^ rad/s	0.54 × 10^−5^ rad/s
	*y*	4.58 × 10^−5^ rad/s	0.84 × 10^−5^ rad/s
	*z*	3.36 × 10^−5^ rad/s	0.35 × 10^−5^ rad/s

**Table 12:** Convergence time of the standard ZUPT and TCZUPT filter in case of residual error of 15 degree/h.

Axis	Standard ZUPT Filter	TCZUPT Filter
*x*	140 s	65 s
*y*	345 s	127 s
*z*	85 s	53 s
